# Hydrogen saline prevents selenite-induced cataract in rats

**Published:** 2013-07-29

**Authors:** Chun-xiao Yang, Hong Yan, Tian-bing Ding

**Affiliations:** 1 Department of Ophthalmology, Tangdu Hospital, Fourth Military Medical University, Xi’an, China;; 2 Department of Microbiology, Fourth Military Medical University, Xi’an, China

## Abstract

**Purpose:**

The aim of this study was to investigate the potential antioxidative effect and mechanism for the protective effects of hydrogen saline on selenite-induced cataract in rats.

**Methods:**

Sprague-Dawley rat pups were divided into the following groups: control (Group A), selenite induced (Group B), and selenite plus hydrogen saline treated (Group C). Rat pups in Groups B and C received a single subcutaneous injection of sodium selenite (25 μmol/kg bodyweight) on postnatal day 12. Group C also received an intraperitoneal injection of H_2_ saline (5 ml/kg bodyweight) daily from postnatal day 8 to postnatal day 17. The development of cataract was assessed weekly by slit-lamp examination for 2 weeks. After sacrifice, extricated lenses were analyzed for activities of superoxide dismutase, catalase, glutathione peroxidase, glutathione reductase, and glutathione S-transferase, levels of malondialdehyde, reduced glutathione (GSH), and total sulfhydryl contents.

**Results:**

The magnitude of lens opacification in Group B was significantly higher than in Group A (p<0.05), while Group C had less opacification than Group B (p<0.05). Compared with Group B, the mean activities of the antioxidant enzymes superoxide dismutase, catalase, glutathione peroxidase, glutathione reductase, and glutathione S-transferase, levels of GSH, and total sulfhydryl contents were higher, whereas the level of malondialdehyde was lower following treatment with hydrogen saline(p<0.05).

**Conclusions:**

This is an initial report showing that hydrogen saline can prevent selenite-induced cataract in rats. It acts via maintaining antioxidant enzymes and GSH, protecting the sulfhydryl group, and inhibiting lipid peroxidation.

## Introduction

Cataract, defined as any opacity in the lens, is the leading cause of blindness around the world, affects up to 80% of the human population over the age of 70, and seriously impairs vision and quality of life [1]. It has increased in prevalence in many countries as a result of a growing elderly population and the incidence of cataract is expected to rise in the future [2]. Currently, the only effective treatment for cataract is surgical removal and replacement of the cataract with an artificial intraocular lens. However, cataract surgery may result in complications such as posterior capsular opacity, glaucoma, endophthalmitis, uveitis, retinal detachment, etc [3]. In addition, the cost of surgery poses an economic burden on patients. Therefore, it is important to explore alternative pharmacological measures for the treatment of cataract.

Cataract is a multifactorial eye disease associated with several risk factors such as oxidative damage, abnormality of glucose metabolism, irradiation damage, and intoxicant damage. Both epidemiological and experimental studies have provided evidence that oxidative stress is a major mechanism in the initiation and progression of cataract [4]. According to the oxidative stress hypothesis of cataract formation, reactive oxygen species (ROS) lead to a surge of detrimental biochemical reactions, including oxidation, crosslinking and aggregation of lens proteins, peroxidation of membrane lipids, and apoptosis of the lens epithelial cells [5]. There exists a group of oxygen eliminators in the lens, including reduced glutathione (GSH), superoxide dismutase (SOD), catalase (CAT), glutathione peroxidase (Gpx), glutathione reductase (GR), and glutathione S-transferase (GST), protecting crystallins from oxidative damage, but their active oxygen-scavenging activities are not strong enough to counteract cataract formation in the lens [6].

Selenite-induced cataract is a cataract model that is causally related to oxidative stress, where oxidation of the critical sulfhydryl groups is essential for the initiation of cataractogenesis [7]. This model is widely used because of its rapidity, easiness, and reproducibility. Various compounds have been shown by experimental studies to prevent selenite-induced cataractogenesis, including ascorbic acid [8], pyruvate [5], resveratrol [9], melatonin [10], ellagic acid [11], carnosine, N-acetylcysteine [12], and onion juice [13]. However, these may generate secondary metabolic reactions that need to be eliminated in vivo, and because their reducibility is so strong, the metabolic oxidation-reduction reactions in the lens may be disturbed.

Recent studies have demonstrated that molecular hydrogen (H_2_), a novel antioxidant, has a therapeutic role in many diseases. Hydrogen does not influence the metabolic oxidation-reduction reactions, innate immune system, or physiologic parameters in vivo [14,15]. Hydrogen gas may selectively reduce hydroxyl radicals and inhibit oxidative stress–induced organ injuries, such as those of the brain, heart, liver, lung, kidney, and pancreas [14,16-20] The administration of hydrogen saline has been shown to reduce retinal ischemia, protect against glutamate-induced retinal injury and hyperoxia-induced retinal neovascularization, inhibit corneal neovascularization caused by alkali burn, and prevent diabetic retinopathy [19,21-24]. It is reasonable and interesting to investigate whether molecular hydrogen has a potential therapeutic value for cataractogenesis. In this study, we tested the effects of intraperitoneal application of hydrogen saline on cataractogenesis in a selenite-induced cataract rat model.

## Methods

### Materials

Sodium selenite was obtained from Sigma Chemical Company (Beijing, China). Protein and enzyme quantification kits were purchased from the Jiancheng Bioengineering Institute (Nanjing, China). Sprague-Dawley rats were provided by the Animal Laboratories of the Fourth Military Medical University (Xi’an, China). All other chemicals and reagents used in biochemical measurement were obtained from local companies.

### Hydrogen saline preparation

For the saturated hydrogen saline preparation, purified H_2_ was dissolved into normal saline for 2 h under 0.6 MPa. The hydrogen-saline was stored under atmospheric pressure at 4 °C in an aluminum bag with no dead volume. Hydrogen saline was freshly prepared every week to maintain a constant concentration of 0.6 mmol/l [25].

### Experimental groups

All experimental procedures were performed in accordance with the Association for Research in Vision and Ophthalmology Statement for the Use of Animals in Ophthalmic and Vision Research and Guidelines on Animal Care and approved by Fourth Military Medical University Animal Protocol Management and Review Committee. Neonatal rat pups of the Sprague-Dawley strain initially weighing 12–18 g on the 7th day of age were used in this study. The pups were housed along with their mother in polypropylene cages under a 12h:12h light-dark cycle, at room temperature (24±1 °C). The animals were maintained on a standard laboratory animal diet and provided water ad libitum.

Cataract was induced in suckling rats at postnatal day 12 by a single subcutaneous injection of sodium selenite (19–30 μmol/kg bodyweight) [7]. The doses of H_2_ saline (5 ml/kg bodyweight) and sodium selenite (25 μmol/kg bodyweight) were based on the results of the preliminary study.

In the preliminary experiments, the rat pups were randomly divided into six groups and labeled as A, B, C, D, E, and F. The doses were as follows:

• Group A: Rat pups received only saline;

• Group B: Rat pups received sodium selenite alone (on the 12th day of age);

• Group C: Rat pups received sodium selenite (on the 12th day of age) and H_2_ saline (from the 8th day up to the 17th day of age);

• Group D: Rat pups received sodium selenite (on the 12th day of age) and H_2_ saline (from the 8th day up to the 12th day of age);

• Group E: Rat pups received sodium selenite (on the 12th day of age) and H_2_ saline (from the 17th day up to the 21st day of age);

• Group F: Rat pups received only H_2_ saline (from the 8th day up to the 17th day of age).

The examination of both eyes of each rat pup was carried out weekly for 2 weeks by slit-lamp microscopy. Rats in Groups A and F exhibited complete transparency of the lens. The extent of lens opacification in Group C was significantly less than in Group B, but the extent of lens opacification in Groups D and E were similar to that in Group B. Therefore, we chose Groups A, B, and C for the following study.

In the study, the rat pups were randomly divided into three groups, each comprising 20 pups, as follows:

• Group A: the untreated normal control group, which received only saline,

• Group B: the untreated model control group, which received sodium selenite alone (cataract-untreated); and

• Group C: the H_2_ saline–treated group, which received sodium selenite and H_2_ saline (cataract-treated).

Each rat pup in Groups B and C received a single subcutaneous injection of sodium selenite (25 μmol/kg bodyweight) on the 12th day of age. Group C received an intraperitoneal injection of H_2_ saline (5 ml/kg bodyweight) daily from postnatal day 8 to postnatal day 17.

### Slit-lamp microscope examination and cataract classification

Cataract could be visualized from the 16th day of age with the naked eye when the rat pups first opened their eyes. The development of cataract was assessed weekly for 2 weeks by slit-lamp examination. At final examination, the pupils were dilated with 0.5% tropicamide solution twice at an interval of 5 min. All eyes of rats were observed under a slit-lamp microscope (Haag-Streit BQ 900 model; Hagg-Streit International, Koeniz, Switzerland) on postnatal day 26, using 40× magnification. Classification of the cataract stages was graded according to the method reported by Hiraoka and Clark [26], briefly described as follows: grade 0, normal transparent lens; grade 1, initial signs of posterior subcapsular or nuclear opacity involving tiny scatters; grade 2, slight nuclear opacity with swollen fibers or posterior subcapsular scattering foci; grade 3, diffuse nuclear opacity with cortical scattering; grade 4, partial nuclear opacity; grade 5, nuclear opacity without lens cortex; and grade 6, mature cataract of the entire lens. The double-blind method was used to classify the degree of lens opacification.

### Preparation of lenses for analysis

All rats were sacrificed with an overdose of anesthesia with chlorpromazine (50 mg/kg) on postnatal day 26. The lenses were removed from eyes that had been excised by a posterior approach. Each lens was weighed, added to nine times its mass (1 g: 9 ml) of 0.9% ice-cold saline (pH=7.2), homogenized by a handheld homogenizer for 15 min over ice, and finally centrifuged at 2,200g for 10 min at 4 °C to obtain a clear supernatant.

### Protein determination

Protein concentration in each sample was determined by Coomassie brilliant blue method by using the protein assay kit from the Jiancheng Bioengineering Institute. The clear supernatant was used for water-soluble protein determination according to the manufacturer’s instructions. The blue complexes were read at 595 nm, and the results were expressed as g/l.

### Assay of antioxidant enzyme activity

#### Superoxide dismutase

SOD activity in lens homogenate was measured with xanthine oxidase method according to instructions of the SOD assay kit [27]. The degree of inhibition of 4-nitro-blue tetrazolium chloride (NBT) using the xanthine-xanthine oxidase system to generate superoxide anions was measured. SOD inhibits auto-oxidation of hydroxylamine. The absorbance values were detected by a spectrophotometer with ultra-micro cuvettes. The wavelength was set at 550 nm and the results were expressed as U/mg protein.

#### Catalase

CAT activity was assayed using the ammonium molybdate method according to instructions of the CAT assay kit [28]. The faint yellow complexes were read at 405 nm. The results were expressed as U/mg protein and 1 U of CAT activity represents 1 μmol H_2_O_2_ decomposition per second.

#### Glutathione peroxidase

Gpx activity was measured according to the instructions of the Gpx assay kit from the Jiancheng Bioengineering Institute. The rate of glutathione oxidation catalyzed by Gpx present in the supernatant was determined, with H_2_O_2_ as a cofactor [29]. The resulting yellow was read spectrophotometrically at 412 nm. The enzyme activity was expressed as U/mg protein. One U of Gpx activity is defined as the amount of enzyme that converts 1 μmol of GSH to the oxidized glutathione in the presence of H_2_O_2_ per minute.

#### Glutathione reductase

GR activity was assayed according to instructions of the GR assay kit from the Jiancheng Bioengineering Institute. The reaction was initiated by the addition of 20 μl of lens homogenates. Oxidized glutathione was reduced to GSH catalyzed by GR with nicotinamide adenine dinucleotide phosphate (NADPH) as a cofactor. The decrease in the optical density was read at 340 nm over 2 min at intervals of 30 s on a spectrophotometer. The enzymatic activity was calculated using an extinction coefficient of 6.22 mM/cm for NADPH [30]. The enzyme activity was expressed as U/g protein. One U of GR activity is equivalent to the oxidation of 1 mmol of NADPH per minute.

#### Glutathione S-transferase

GST activity was measured according to instructions of the GST assay kit from the Jiancheng Bioengineering Institute. The conjugation of GSH with 1-chloro, 2–4 dinitrobenzene, a hydrophilic substrate, was observed spectrophotometrically at 412 nm to measure the activity of GST [31]. The enzyme activity was expressed as U/mg protein. One U of GST activity is defined as the amount of enzyme required to conjugate 1 μmol of 1-chloro, 2–4 dinitrobenzene with GSH per minute.

### Determination of malondialdehyde content

The content of malondialdehyde (MDA) was determined by thiobarbituric acid reaction chromometry with reference to the MDA assay kit from the Jiancheng Bioengineering Institute. The intensity of the resulting pink color was read at 532 nm [32]. The level of lipid peroxide is expressed as nmol of MDA formed per mg protein.

### Determination of reduced glutathione and total sulfhydryl content

The GSH content in each lens was determined with 5, 5′-dithiobis-nitrobenzoic acid, using the 10% trichloroacetic acid–soluble fraction of the supernatants. The absorbance of the resulting yellow color was measured using a spectrophotometer at 420 nm. The results were expressed as μmol/g protein.

The total sulfhydryl content was determined using the total sulfhydryl assay kit from Jiancheng Bioengineering Institute according to the manufacturer’s instructions. The yellow compound yielded by the reaction of sulfhydryls and 5, 5′-dithiobis-nitrobenzoic acid exhibited highest absorption of light at 412 nm on a spectrophotometer [33]. The results were expressed as μmol/g protein.

### Statistical analysis

All data were expressed as the mean± standard deviation (SD). Statistical analysis of the data was subject to one-way analysis of variance (ANOVA) followed by the least significant difference (LSD) test. The chi-square test was applied for the categorical variables. All statistical calculations were carried out using the Statistical Package for Social Science version 18.0 (SPSS, Chicago, IL). The significance level was set at p<0.05.

## Results

### Lens opacification observed under slit-lamp microscope

**Figure 1:**
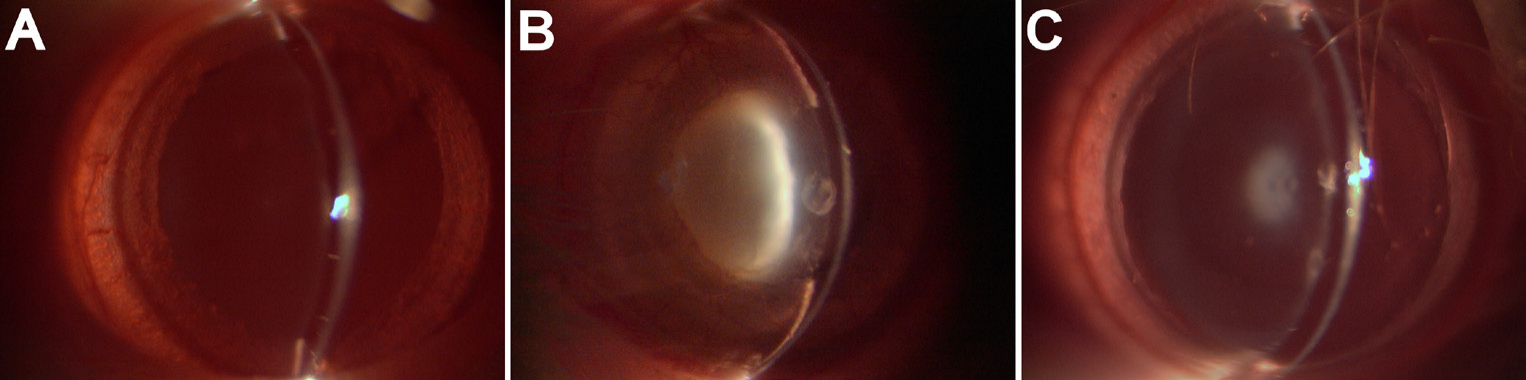
Lens opacification in the eyes of the 26-day-old rat pups under slit-lamp microscope (magnification of 40×) in various experimental groups. **A**: Rat pups received only saline (Group A). **B**: Rat pups received only selenite (Group B). **C**: Rat pups received selenite and H_2_ saline (Group C).

At the final examination on postnatal day 26, the pups were evaluated for cataract development and photographed. Different grades of selenite cataracts are demonstrated in Table 1 and Figure 1. Group A rats that received normal saline exhibited complete transparency of the lens. Subcutaneous injections of sodium selenite resulted in lens opacities in all eyes in Group B. Among these, 20% of the eyes developed partial nuclear opacity (Grade IV), 25% developed nuclear opacity (Grade V), and 55% developed mature cataract of the entire lens (Grade VI). In contrast, in the H_2_ saline injection group (Group C, 5 ml/kg), 5% of the eyes developed slight nuclear opacity (Grade II), 25% developed diffuse nuclear opacity (Grade III), 45% developed partial nuclear opacity (Grade IV), 15% developed nuclear opacity (Grade V), and 10% developed mature cataract of the entire lens (Grade VI). There was a statistically significant difference between Group C and Group B (p<0.05).

**Table 1:** Slit-lamp microscope examination and cataract classification.

**Experimental** **groups**	**Number of rats**	**0**	**I**	**II**	**III**	**IV**	**V**	**VI**
Group A	40	40 (100%)						
Group B*	40					8(20%)	10(25%)	22(55%)
Group C**^#^**	40			2(5%)	10(25%)	18(45%)	6(15%)	4(10%)

Data are presented as N(%); Statistics analysis was by χ2 test; The degree of opacification was graded as follows: grade 0, normal transparent lens; grade 1, the initial sign of posterior subcapsular or nuclear opacity involving tiny scatters; grade 2, the slight nuclear opacity with swollen fibers or posterior subcapsular scattering foci; grade 3, the diffuse nuclear opacity with cortical scattering; grade 4, the partial nuclear opacity; grade 5, nuclear opacity without lens cortex; grade 6, mature cataract of entire lens. Group A: Rat pups received only saline. Group B: Rat pups received only selenite. Group C: Rat pups received selenite and hydrogen-saline. * Compared with Group A: p<0.05. # Compared with Group B: p<0.05.

### Protein determination

**Figure 2:**
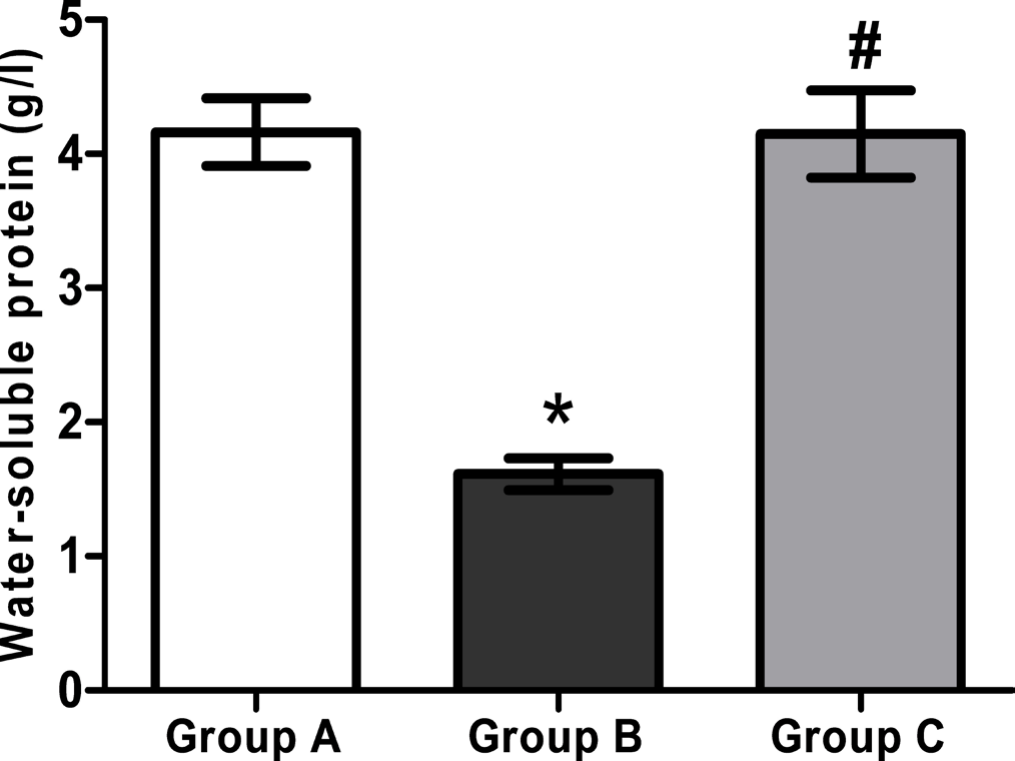
The concentrations of water-soluble proteins in lenticular samples of the 26-day-old rat pups in various experimental groups. Group A: Rat pups received only saline. Group B: Rat pups received only selenite. Group C: Rat pups received selenite and hydrogen saline. Values are expressed as mean±standard deviation (SD). Statistical analysis of the data was subject to one-way analysis of variance (ANOVA) followed by the least significant difference (LSD) test. *Group B is compared with Group A: p<0.05. # Group C is compared with Group B: p<0.05 (n=9).

There was a significant (p<0.05) decline in water-soluble protein contents in Group B (1.61±0.12 g/l) compared to Group A (4.16±0.25 g/l). Treatment with H_2_ saline augmented the water-soluble protein level, as a statistically significant difference was shown between Group C (4.15±0.33 g/l) and Group B (p<0.05; Figure 2).

### Activities of antioxidant enzymes

#### Superoxide dismutase

**Figure 3:**
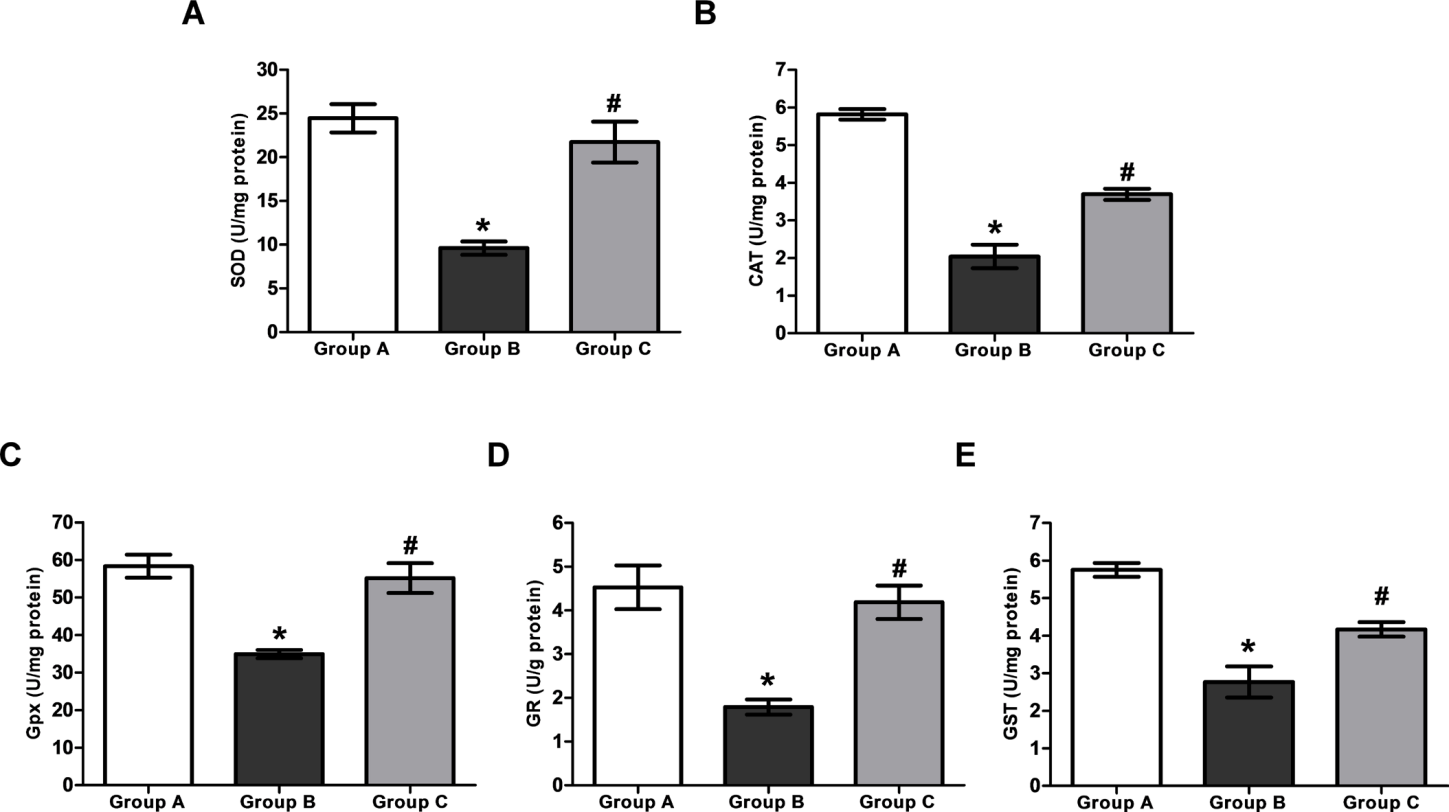
The activities of antioxidant enzymes in lenticular samples of the 26-day-old rat pups in various experimental groups. Group A: Rat pups received only saline. Group B: Rat pups received only selenite. Group C: Rat pups received selenite and hydrogen saline. **A**: The activity of superoxide dismutase (SOD) in lens (n=9). **B**: The activity of catalase (CAT) in lens (n=9). **C**: The activity of glutathione peroxidase (Gpx) in lens (n=6). **D**: The activity of glutathione reductase (GR) in lens (n=9). **E**: The activity of glutathione S- transferase (GST) in lens (n=9). Values are expressed as mean±standard deviation (SD). Statistical analysis of the data was subject to one-way analysis of variance (ANOVA) followed by the least significant difference (LSD) test. * Compared with Group A: p<0.05. # Compared with Group B: p<0.05.

The mean activity of SOD in Group B (9.60±0.76 U/mg protein) was significantly (p<0.05) lower than that of in Group A (24.45±1.61 U/mg protein), while treatment with H_2_ saline in Group C restored SOD activity. SOD activity in Group C (21.73±2.34 U/mg protein) was found to be significantly higher (p<0.05) than that in Group B (Figure 3A).

#### Catalase

A significantly (p<0.05) lower mean activity of CAT was observed in Group B (2.04±0.31 U/mg protein) when compared with Group A (5.82±0.14 U/mg protein). H_2_ saline treatment resulted in a significant (p<0.05) increase of CAT activity in Group C (3.69±0.15 U/mg protein) compared to Group B (Figure 3B).

#### Glutathione peroxidase

The administration of selenite, which induced oxidative stress in the lenses, resulted in significantly (p<0.05) reduced activity of Gpx in Group B (34.90±1.11 U/mg protein) compared with that in Group A (58.31±3.06 U/mg protein), but this reduction was reversed to a near-normal level in Group C (55.15±3.98 U/mg protein) by H_2_ saline (p<0.05; Figure 3C).

#### Glutathione reductase

The activity of GR in Group B (1.79±0.17 U/g protein) was significantly (p<0.05) lower than that in Group A (4.53±0.50 U/g protein). The enzymatic activity showed that selenite damaged the activity of GR in the lenses of rat pups. The reduction of GR activity was almost reversed (p<0.05) by H_2_ saline treatment in Group C (4.19±0.38 U/g protein; Figure 3D).

#### Glutathione S-transferase

The activity of GST was reduced significantly (p<0.05) in Group B (2.77±0.41 U/mg protein) in comparison with that in Group A (5.75±0.18 U/mg protein). GST activity was notably recovered by H_2_ saline treatment in Group C (4.17±0.19 U/mg protein) as compared with Group B (p<0.05; Figure 3E).

### Levels of malondialdehyde content

**Figure 4:**
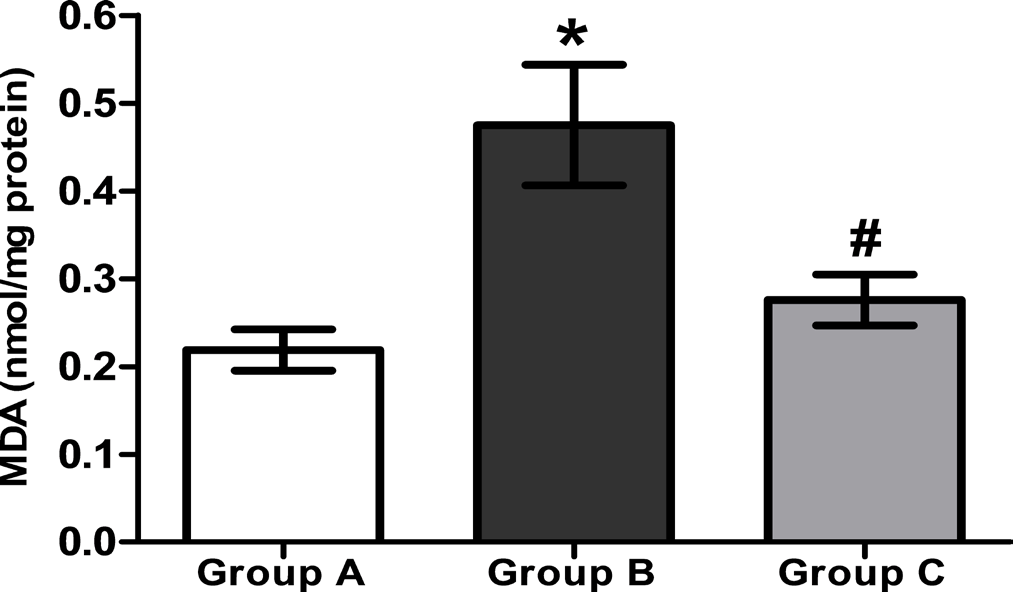
The level of malondialdehyde (a lipid peroxidation product index) in lenticular samples of the 26-day-old rat pups in various experimental groups. Group A: Rat pups received only saline. Group B: Rat pups received only selenite. Group C: Rat pups received selenite and hydrogen saline. Values are expressed as mean±standard deviation (SD). Statistical analysis of the data was subject to one-way analysis of variance (ANOVA) followed by the least significant difference (LSD) test. * Compared with Group A: p<0.05. # Compared with Group B: p<0.05 (n=8).

MDA, an indicator of lipid peroxidation, was significantly (p<0.05) increased in Group B (0.47±0.07 nmol/mg protein) compared with Group A (0.22±0.02 nmol/mg protein). The mean concentration of MDA in Group C (0.28±0.03 nmol/mg protein) was significantly (p<0.05) lower than that in Group B (Figure 4). The results suggested that H_2_ saline possibly protected the structural integrity of lenticular membrane lipids, thereby preventing opacification of the lens.

### Levels of reduced glutathione and total sulfhydryl contents

**Figure 5:**
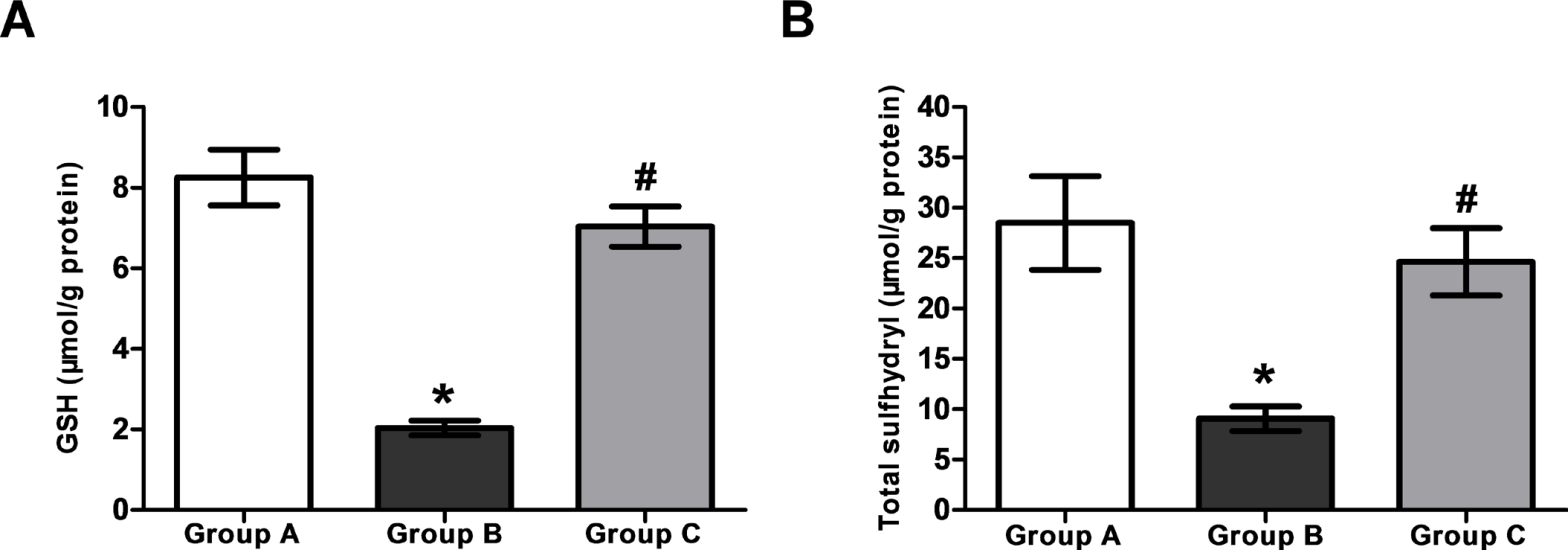
The levels of reduced glutathione and sulfhydryl content in lens. **A**: The levels of reduced glutathione in lenticular samples of the 26-day-old rat pups in various experimental groups (n=6). **B**: The levels of total sulfhydryl content in lenticular samples of the 26-day-old rat pups in various experimental groups (n=6). Group A: Rat pups received only saline. Group B: Rat pups received only selenite. Group C: Rat pups received selenite and hydrogen saline. Values are expressed as mean±standard deviation (SD). Statistical analysis of the data was subject to one-way analysis of variance (ANOVA) followed by the least significant difference (LSD) test. * Compared with Group A: p<0.05. # Compared with Group B: p<0.05.

Selenite administration resulted in a significant (p<0.05) reduction of GSH in the concentration in comparison with Group A (8.26±0.69 μmol/g protein), whereas the treatment with H_2_ saline in Group C (7.04±0.50 μmol/g protein) was found to maintain a significantly (p<0.05) higher level of GSH concentration compared to Group B (2.04±0.18 μmol/g protein; Figure 5A).

Compared with Group A (28.50±4.66 μmol/g protein), Group B (9.08±1.22 μmol/g protein) exhibited a significant (p<0.05) decrease in total sulfhydryl contents. In contrast, the total sulfhydryl contents in Group C (24.66±3.33 μmol/g protein) were significantly (p<0.05) higher than in Group B (Figure 5B). All of the above data indicated that the antioxidant capacity of H_2_ saline may be involved in both nonenzymatic and enzymatic antioxidant systems.

## Discussion

This study represents an early attempt to evaluate the potential therapeutic value of hydrogen saline (H_2_) against cataract formation. The results showed that hydrogen saline could reduce cataract formation and restore antioxidant capacity in the selenite cataract model. These protective effects included maintaining water-soluble protein contents, GSH level, total sulfhydryl groups, and antioxidant enzymes activities, and reducing MDA accumulation in the lenses of selenite-treated rat pups.

Selenite-induced cataract has received much attention as a model system for oxidative stress–induced cataract. It is similar to human cataract in many aspects, including increased calcium, protein aggregation, decreased water-soluble protein, and reduced glutathione levels [34]. Oxygen free radicals disturb cellular homeostasis through the modification of proteins and lipid peroxidation. The lens has a well-designed system of defense against oxidation. Selenite causes oxidation of protein and nonprotein sulfhydryl groups, which leads to ion pump damage and disturbance of the electrolytic balance. The intracellular calcium level increases, which activates a protease—calpain—resulting in the partial hydrolysis of intracellular proteins, especially lens β-crystallin. Assembled protein aggregates scatter light, and thus increase opacity, which is accompanied by a decrease in activities of antioxidant enzymes [9,10]. Primary defenses, including nonenzymatic antioxidants and enzymatic antioxidants such as glutathione, SOD, CAT, Gpx, GR, and GST, neutralize free radicals and repair, recover, or degrade molecules that are damaged [7]. Free radicals also react with the protein-thiol groups, leading to crosslinking and protein aggregation until the increase of water-insoluble proteins finally results in the formation of cataract [35].

The antioxidant enzymes are able to catalytically remove free radicals and other reactive species. A wide array of enzymatic antioxidant defenses exists, including SOD, CAT, Gpx, GR, and GST. SOD exists in two forms, one containing Mn^2+^, confined to the mitochondria, and a cytosolic form containing Zn^2+^ and Cu^2+^. It converts superoxide to H_2_O_2_. The two-electron dismutation of H_2_O_2_ is catalyzed into ground-state oxygen and water by CAT and enzymes of the glutathione redox cycle, including GR and Gpx [36]. Gpx is the predominant GSH-consuming enzyme, and the Gpx family uses GSH as a cofactor to destroy H_2_O_2_ and lipoperoxides at low levels of H_2_O_2_; at a higher concentration, the principal mechanism for the removal of H_2_O_2_ is CAT [37]. GR is the rate-controlling enzyme of the glutathione redox cycle, and the intracellular level of GSH is maintained by GR via preserving the integrity of cell membranes and stabilizing the sulfhydryl groups of proteins [38,39]. GST, a typical multifunctional enzyme, is viewed as a defense mechanism against lipid peroxidation, and plays a role in the hydrophobic compounds as a thioltransferase-like redox regulator [40].

In our experiments, the mean activities of SOD, CAT, Gpx, GR, and GST significantly decreased in the lenses of the untreated selenite-injected group (Group B) compared with the normal control group (Group A). In rat pups treated with hydrogen saline (Group C), the mean activities of antioxidant enzymes were largely restored when compared with the lenses in Group B. Hydrogen saline modulates several biological functions, and exhibits anti-inflammatory and antioxidant activities. The ability of hydrogen saline to neutralize free radicals, especially the hydroxyl radicals and other important ROS, has been reported under conditions of hyperoxia-induced oxidative stress [24]. In addition, elevated activities of antioxidant enzymes have been reported in senescence-accelerated mice [41].

GSH, a major nonprotein thiol, is a vital intra- and extracellular antioxidant that protects against oxidative stress. It exists in a high concentration in the lens and is important for sustaining lens proteins in a reduced state through its redox and detoxification reactions [42]. Composed of cysteine, glutamic acid, and glycine, GSH protects the lens from oxidative damage and maintains the transparency of the lens, allowing it to perform normal functions by protecting the protein sulfhydryl groups from oxidation [43]. In selenite-induced cataract, the depletion of GSH occurs through a nonenzymatic reaction of GSH with selenite to form a derivative, selenodiglutathione (GS-Se-SG), giving rise to the formation of superoxide anion as an intermediate [44]. Sulfhydryl oxidation is demonstrated to be one of the major pathological events leading to disulfide crosslinking and molecular aggregation, and through this, to protein precipitation and lens opacification [45]. Lipid peroxidation has been strongly implicated in the mechanism of cataractogenesis. MDA, a product of lipid peroxidation, is accepted as a reliable marker of the lipid peroxidation that occurs because of oxidative stress [46].

In the present study, when the rat pups were treated with hydrogen saline (Group C), the mean GSH and total sulfhydryl were found to be significantly higher than those in the lenses of the untreated cataract group (Group B). These findings corroborate an earlier report demonstrating that hydrogen saline significantly restored GSH levels in rats with oxidative stress–induced damage in skeletal muscle after acute exhaustive exercise [47]. The enhancement of GSH levels can be included among the beneficial effects of hydrogen saline treatment, and may be due to a higher expression of the enzymes involved in glutathione synthesis. The observed increase of the MDA level in the lenses of the untreated model control group (Group B) compared with the untreated normal control group (Group A) may account for the disruption of membrane lipids. In addition, the reduction of the MDA level in hydrogen saline treated group (Group C) suggests that hydrogen saline possibly prevented the disruption of lenticular membrane lipids, thereby impeding opacification of the lens. Similar findings regarding the effects of hydrogen saline on lipid peroxidation were reported by Huang et al. in relation to hyperoxia-induced retinopathy [24].

Therapeutic antioxidant medical gas may be a reasonable approach for the treatment of oxidative stress [48]. Hydrogen is one very promising gaseous agent that has come to the forefront of research over the last few years. Hydrogen saline has been proved to have antioxidant properties both in vitro and in vivo [49]. It is superior to hydrogen gas because higher concentrations of hydrogen can be dissolved, and hydrogen saline is safer and easier to handle [50]. There is accumulating evidence that hydrogen saline reduces oxidative injury in various disease models [19,21-24]. Hydrogen can easily penetrate biomembranes and diffuse into the cytosol, mitochondria, and nucleus due to its small molecular weight [17]. Hydrogen specifically scavenges hydroxyl radicals, peroxynitrites, and other important ROS, e.g., H_2_O_2_ and O_2_^-^, both of which are extremely reactive and damaging to nucleic acids, lipids, and proteins. H_2_ decreases hydroxyl radicals levels in the nuclear region and reduces hydroxyl radicals, but does not affect •O_2_^-^; H_2_O_2_ has its own physiological roles. In the presence of catalytically active metals, however, detoxification of superoxide to H_2_O_2_ by SOD generates more potent hydroxyl radicals.

Ohsawa drew the conclusion that hydrogen gas protects cells from oxidative damage through selective scavenging of hydroxyl radicals and peroxynitrites [14]. In addition, hydrogen does not disturb the metabolic oxidation-reduction reactions or the innate immune system [14]. It has also been found that hydrogen does not influence physiological parameters (temperature, blood pressure, pH, partial pressure of oxygen[ pO_2_]) [15]. Hydrogen has many advantages from the aspect of toxicity, as it has no cytotoxicity even at high concentrations. Furthermore, safety standards have been established for high concentrations of hydrogen gas for inhalation, since high-pressure hydrogen gas is used in deep diving gas mixes to prevent decompression sickness and arterial gas thrombi. The safety of hydrogen for humans has been demonstrated by its application in Hydreliox, an exotic breathing gas mixture of 49% H_2_, 50% helium and 1% O_2_, which has been used to prevent decompression sickness and nitrogen narcosis during very deep technical diving [51-54]. The tissue compatibility of hydrogen is better than that of many other antioxidants. Moreover, compared to other drugs, the cost of hydrogen therapy is much lower.

In conclusion, the data from our experiments demonstrated that hydrogen saline effectively retarded selenite-induced cataract formation. As an antioxidant agent, hydrogen saline increased the levels of GSH, protected the sulfhydryl groups, maintained antioxidant enzyme activities, and inhibited lipid peroxidation, thus sustaining lens transparency. Furthermore, hydrogen saline appears to be a potential therapy with advantages of less expense and safer implementation in many clinical settings.

## 
